# U-Net-Based Fingerprint Enhancement for 3D Fingerprint Recognition

**DOI:** 10.3390/s25051384

**Published:** 2025-02-24

**Authors:** Mohammad Mogharen Askarin, Min Wang, Xuefei Yin, Xiuping Jia, Jiankun Hu

**Affiliations:** 1 School of Systems and Computing, University of New South Wales, Canberra, ACT 2612, Australia; m.mogharen_askarin@unsw.edu.au (M.M.A.); min.wang@canberra.edu.au (M.W.); 2 School of Information Technology and Systems, University of Canberra, Canberra, ACT 2617, Australia; 3 School of Information and Communication Technology, Griffith University, Brisbane, QLD 4222, Australia; x.yin@griffith.edu.au; 4 School of Engineering and Technology, University of New South Wales, Canberra, ACT 2612, Australia; x.jia@unsw.edu.au

**Keywords:** biometrics, fingerprint, 3D, deep learning

## Abstract

Biometrics-based authentication mechanisms can address the built-in weakness of conventional password or token-based authentication in identifying genuine users. However, 2D-based fingerprint biometrics authentication faces the problem of sensor spoofing attacks. In addition, most 2D fingerprint sensors are contact-based, which can boost the spread of deadly diseases such as the COVID-19 virus. Three-dimensional fingerprint-based recognition is the emerging technology that can effectively address the above issues. A 3D fingerprint is captured contactlessly and can be represented by a 3D point cloud, which is strong against sensor spoofing attacks. To apply conventional 2D fingerprint recognition methods to 3D fingerprints, the 3D point cloud needs to be converted into a 2D gray-scale image. However, the contrast of the generated image is often not of good quality for direct matching. In this work, we propose an image segmentation approach using the deep learning U-Net to enhance the fingerprint contrast. The enhanced fingerprint images are then used for conventional fingerprint recognition. By applying the proposed method, the fingerprint recognition Equal Error Rate (EER) in experiment A and B improved from 41.32% and 41.97% to 13.96 and 12.49%, respectively, over the public dataset.

## 1. Introduction

Biometrics-based authentication mechanisms can address the built-in weakness of conventional password or token-based authentication in identifying genuine users [1]. However, a 2D-based fingerprint biometrics authentication system faces the problem of sensor spoofing attacks, where attackers can capture the latent fingerprints left unintentionally on glasses or other objects and then make rubber fingerprints to spoof the 2D fingerprint sensor [2,3,4].

A 3D fingerprint is captured contactlessly and can be represented by a 3D point cloud, which is strong against sensor spoofing attacks. It also enjoys the benefit of being hygienic. However, how to make use of the 3D fingerprint point cloud for effective identity authentication is very challenging. Ding et al. [5] utilized surface and subsurface fingerprint information to enhance authentication accuracy while also making the system more resistant to forgery. He et al. [6] proposed estimating 3D finger angles from a 2D fingerprint image as a method to enhance fingerprint matching accuracy, with potential applications in forensic analysis. To facilitate the study on 3D fingerprints, Dong and Kumar [7] synthesized contactless 3D fingerprints and a new framework to match contactless to contact-based fingerprints. Three-dimensional fingerprints are strong against sensor spoofing attacks as it is infeasible to capture a 3D fingerprint left unintentionally anywhere.

The 3D fingerprint is a three-dimensional representation of a finger surface [8]. Three-dimensional fingerprints can be presented by a 3D point cloud, which is a collection of data points representing the surface of the finger that can be used to extract fingerprint features [9]. In contrast to 2D fingerprints, which only include the surface pattern of a fingerprint, the depth and curvature of ridges can also be extracted from a 3D fingerprint, which offers a higher level of security and spoof resistance [1].

In 2D fingerprint recognition, there are numerous situations where image quality is insufficient for reliable feature extraction, highlighting the necessity for studies on effective image enhancement methods. Ai and Kwon [10] proposed a U-Net enhancement method to increase image contrast for the images taken from surveillance cameras in very-low-light conditions. Huang et al. [11] proposed a latent fingerprint enhancement method by using U-Net architecture, where the model is trained progressively, starting with lower-quality images and gradually moving to higher-quality images. Liu and Qian [12] used a nested U-Net for latent fingerprint enhancement, which uses synthetic latent fingerprints for training. Gavas and Namboodiri [13] proposed a modification of U-Net to enhance low-quality fingerprints.

Three-dimensional fingerprint recognition involves capturing the fingerprint features from a 3D point cloud. One approach that simplifies 3D fingerprint recognition is to unwrap a 3D fingerprint to a 2D image [14] or flatten a 3D fingerprint [15] and then use a conventional 2D fingerprint recognition algorithm for the recognition. The resulting images from these methods may lack sufficient contrast or contain distorted sections, making them unsuitable for direct use in 2D-based fingerprint recognition. Unlike standard 2D fingerprint images, those generated from a flattened 3D point cloud are synthetic, with intensities derived from the relative height of points in the cloud. Currently, no existing image enhancement methods specifically address this scenario.

In this study, we propose an image enhancement method by using a deep U-Net to improve the quality of flattened point cloud images. Note that the flattened point cloud images are fictitious because the gray intensity is not formed from the natural fingerprint ridge–valley structure as in most 2D fingerprint sensors. This work will be the first of its kind in the field.

The rest of this paper is organized as follows: Section 2 describes the method used for flattening the 3D point cloud and our proposed method for fingerprint enhancement. Section 3 details a setup for experiments and the results, and the conclusion of this work is presented in Section 4.

## 2. Methodology

In this section, we first introduce a 3D point cloud flattening method, adapted from an existing 3D fingerprint unwrapping approach, which we use to convert 3D fingerprints into 2D gray-scale fingerprint images. Subsequently, we present the proposed method aimed at enhancing these generated images. Algorithm 1 given below shows an overview of the proposed method.
**Algorithm 1:** Processes used to enhance contrast of the generated gray-scale fingerprint image**Data**: 3D point cloud**Result**: Enhanced gray-scale fingerprint image1Flattening 3D point cloud by cylinder surface fitting2Converting flattened 3D point clouds to 2D gray-scale images to generate training input3Generating ground truth 2D images4Training the U-Net model5Applying the trained model to enhance fingerprint images6**return** *Enhanced fingerprint images*;

### 2.1. Flattening 3D Fingerprint and Generating Gray-Scale Image

The flattening scheme used in our method is derived from a 3D point cloud unwrapping method that uses surface fitting [14]. In this method, the surface of a 3D fingerprint is modeled as the surface of a cylinder. We use this approach to fit a surface to the point cloud. However, instead of unwrapping the data, we flatten the 3D point cloud. This is achieved by subtracting the z-value of each point on the fitted surface from the z-value of its corresponding point in the point cloud.

**Figure 1:**
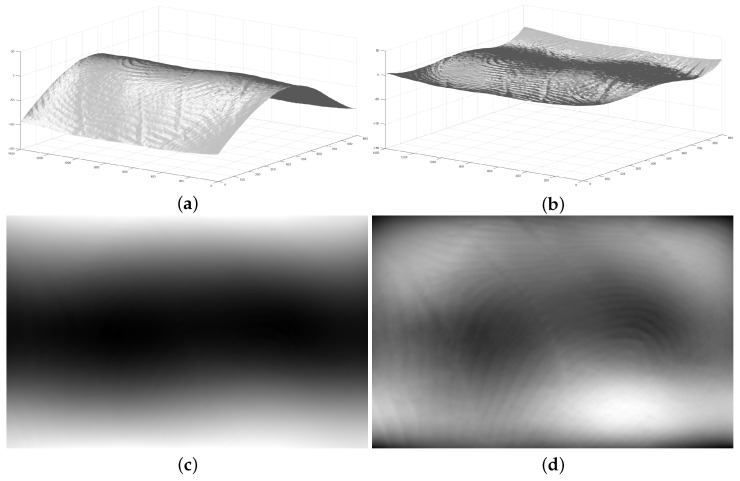
(**a**) A sample 3D fingerprint point cloud. (**b**) The flattened 3D point cloud by using cylinder surface fitting [14]. (**c**) A generated gray-scale image from the sample 3D fingerprint point cloud in (**a**). (**d**) The generated gray-scale image from the flattened 3D point cloud in (**b**).

To generate the fitted surface, the radius of the cylinder needs to be calculated. First, the length of the point cloud is segmented into slices, each with a width of one point. The central slice is then used to determine the radius of the cylinder. By using the chord length (width of the slice) and the arc height (the difference between the z-values of the highest and lowest points in the slice), the radius of the cylinder can be estimated, following a method similar to the one detailed in [16]. Figure 1a shows a sample 3D point cloud and Figure 1b shows the flattened point cloud by applying this concept.

By applying the method described in [15], a flattened 3D point cloud can be converted into a gray-scale image. In this approach, the height of the points (Z value) in the point cloud is used to determine the intensity levels of the corresponding pixels at the same X and Y locations in the gray-scale image. The point with the highest Z value is assigned an intensity of 0, while the point with the lowest Z value is assigned the highest intensity. Points with Z values in between are mapped to intensities ranging from 0 to 255 proportionally. In this way, pixels with higher intensities represent valleys and those with lower intensities represent ridges on the fingerprint surface. Figure 1c,d show the generated gray-scale images from the point clouds depicted in Figure 1a and Figure 1b, respectively. It is obvious that these images are of poor quality and are not suitable for direct matching. We will present our solution for the image enhancement, which will be described in the following.

### 2.2. Fingerprint Enhancement by U-Net

This section describes our proposed method and the preliminary approaches employed for image enhancement using U-Net.

#### 2.2.1. U-Net-Based Full Image Fingerprint Enhancement

**Figure 2:**
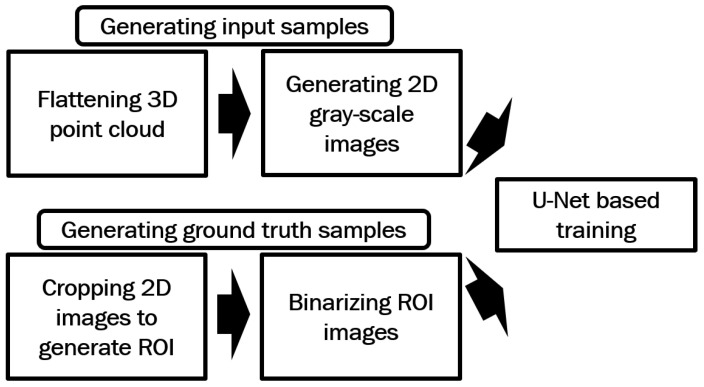
Processes used in the proposed method for generating the training data for U-Net-based full image training.

In this method, the gray-scale images generated in Section 2.1, along with their corresponding ground truth images, are first used to train a U-Net model. The trained model is then applied to enhance the gray-scale fingerprints. Figure 2 illustrates the processes involved in the proposed method for generating training data.

For this purpose, the Hong Kong Polytechnic University 3D Fingerprint Images Database Version 2 [17,18] was utilized. Fingerprint impressions from the first 210 subjects in session 1 of this dataset were used to train the model. The input data for training were generated by converting the point clouds of these 210 subjects into gray-scale images. Each subject has six 3D point cloud impressions, resulting in a total of 1260 gray-scale images used as training input.

**Figure 3:**
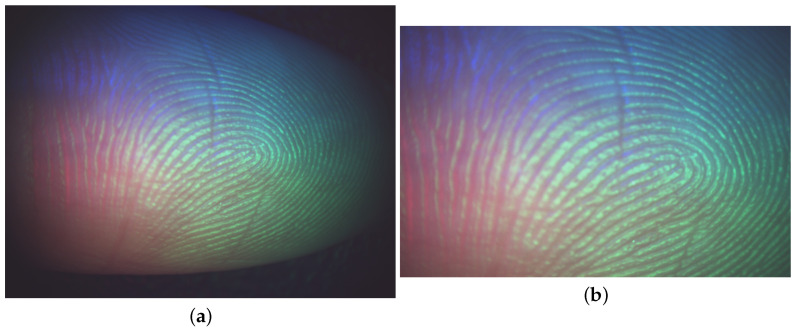
A sample 2D image and its corresponding ROI are shown in (**a**) and (**b**), respectively.

For each point cloud in Dataset A, there are two corresponding contactless 2D fingerprints, each captured under different illumination conditions and originally used to generate the 3D point cloud data. However, these 2D images cannot be directly used as ground truth due to size differences with their 3D point cloud counterparts. In our experiments, the Region of Interest (ROI) of the 2D images was manually selected and cropped by detecting mutual minutiae between the 2D images generated from the 3D point clouds and the contactless 2D images. Figure 3 illustrates a sample contactless 2D image and its corresponding ROI.

**Figure 4:**
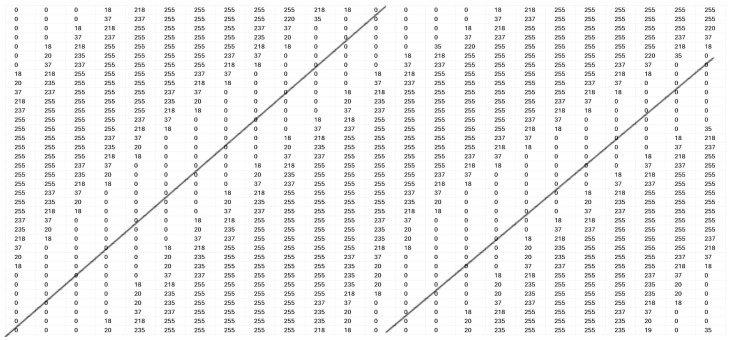
A sample intensity pattern of a section of a binarized image ranging from 0 to 255. The black lines show the position of the ridge patterns and the area in the middle of the black lines shows the position of the valley.

Next, the ROI images were binarized by using VeriFinger [19]. Figure 4 shows the intensity of a section of a binarized image ranging from 0 to 255. The black lines show the position of the ridge patterns and the area in the middle of the black lines shows the position of the valley.

**Figure 5:**
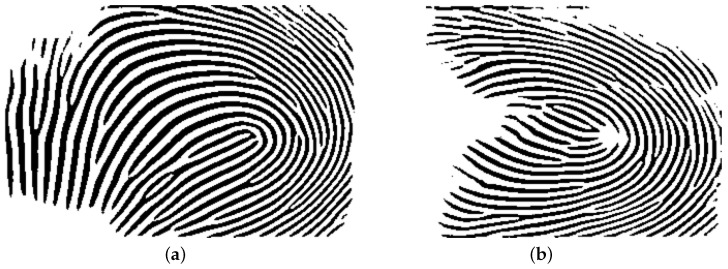
A sample binarized image from Figure 3a shown in (**a**). (**b**) A sample binarized image with blank sections.

Figure 5a shows the binarized image from the ROI image in Figure 3b. The binarization process for some impressions results in blank sections. Figure 5b shows a sample binarized image with such blank areas. The binarized images generated for all the ROI images, totaling 1260 images, will be used as ground truth for training the model.

**Figure 6:**
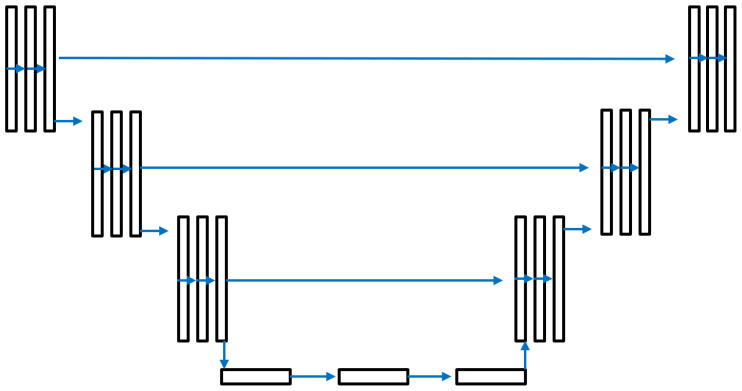
The U-Net model that we used to enhance fingerprint images.

**Figure 7:**
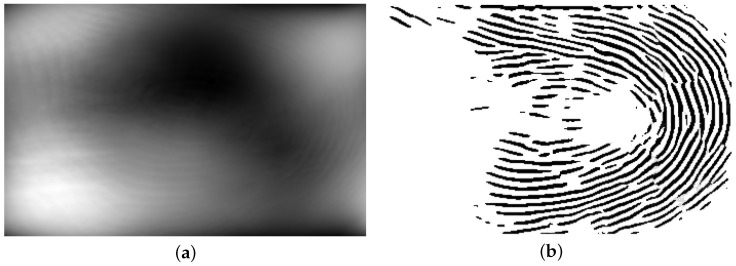
(**a**) A sample generated gray-scale image, and (**b**) the enhanced image using U-Net-based full image fingerprint enhancement.

The U-Net enhancement model is illustrated in Figure 6 with an input and output image size of 352 × 248 pixels. The model includes 256 classes to cover all potential intensity levels in a gray-scale image. The training parameters are shown in Table 1. Figure 7a shows a sample generated gray-scale image, and Figure 7b displays the enhanced image obtained using U-Net-based full image fingerprint enhancement. It can be observed that the visibility of ridge and valley patterns in the enhanced image is significantly improved. Additionally, ridge and valley patterns in some sections that were previously difficult to detect are now clearly visible. However, the enhanced image contains some blank areas, which may result from blank regions in certain ground truth images, as shown in Figure 5b.

#### 2.2.2. Patch-Based Fingerprint Enhancement Using a U-Net Model

In this method, in order the solve the problem of blank areas in the enhanced images in the previous method, we are going to use a patch-based approach. The U-Net enhancement model shown in Figure 6 is also used here, with an image input and output size of 64 by 64 pixels. The same as the previous method, the first 210 subjects in Hong Kong Polytechnic University 3D Fingerprint Images Database Version 2 session one were utilized to train the model.

**Figure 8:**
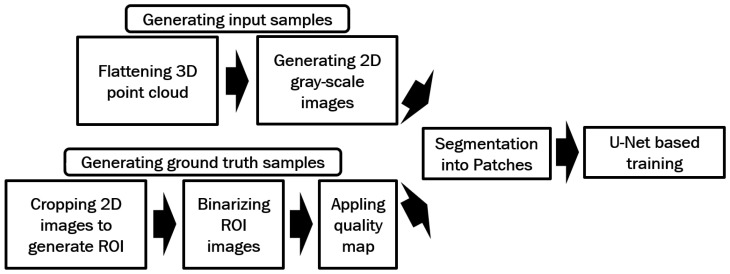
Processes used in the proposed method for generating the training data for patch-based U-Net training.

Figure 8 illustrates the steps involved in the proposed method for generating training data. Following a similar method to the previous approach, 3D point clouds are flattened and transformed into 2D gray-scale images, with corresponding binarized 2D images subsequently generated. However, the binarization process for the ROI images in this method differs as follows:

1. The entire ROI image is initially binarized using VeriFinger.
2. Each individual color channel (e.g., red, green, and blue) of the ROI image is separately binarized using VeriFinger.
3. By using Matlab, the four binarized outputs (one for the full image and three for the color channels) are merged.

**Figure 9:**
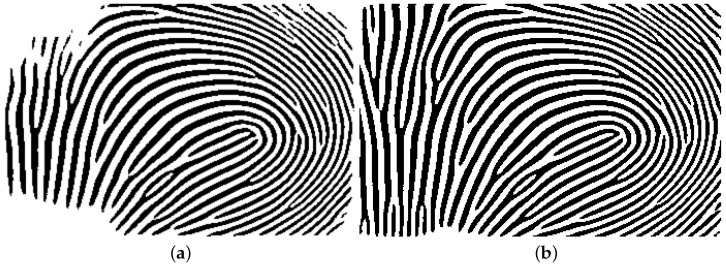
A comparison of the binarized ROI images obtained using the method described in Section 2.2.1 and those generated in Section 2.2.2 is shown in (**a**) and (**b**), respectively.

To ensure proper alignment and avoid overlaps between ridge and valley patterns, only the blank areas (regions without ridge or valley patterns) from one binarized image are merged with the others. Figure 9 compares the binarized ROI images obtained using the previous method with those generated by the proposed method in this section in Figure 9a and Figure 9b, respectively.

**Figure 10:**
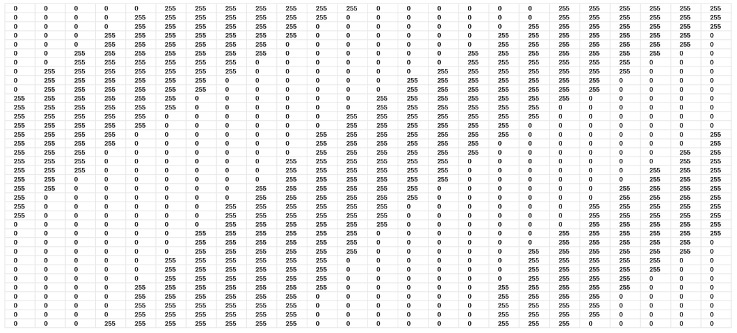
The intensity pattern in a section of a sample binarized ROI image is shown.

The intensity levels in the binarized images are also shifted to two values of 0 and 255 by using Matlab, with 0 indicating a ridge position and 255 indicating a valley position. Figure 10 shows the intensity pattern in a section of a sample binarized ROI image.

**Figure 11:**
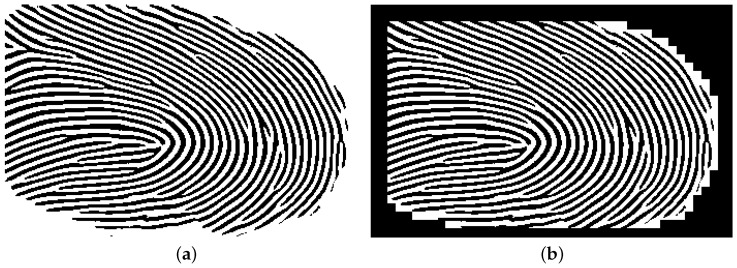
A sample binarized image is shown in (**a**), and its quality-mapped image by excluding lowest-quality sections (quality 0, and quality 1) is shown in (**b**).

Next, MINDTCT [20] is applied to the binarized images, assigning quality scores from 0 to 4 to blocks of 8 × 8 pixels in the fingerprint image. Blocks with a quality score of 0 represent the lowest-quality sections, while blocks with a quality score of 4 indicate the highest-quality sections. The lowest-quality sections (quality 0 and quality 1) are excluded. Figure 11a shows a sample binarized image, and its quality-mapped image by excluding lowest-quality sections (quality 0, and quality 1) is shown in Figure 11b.

The remaining sections of the binarized images are cropped into 64 × 64 pixel patches, which serve as ground truth patches. The corresponding input patches for each ground truth patch are generated by segmenting the 2D gray-scale images of the flattened point clouds, aligned with the locations of the ground truth patches. A total of 9106 input patches and 9106 ground truth patches were used for training the U-Net model. The training parameters are shown in Table 2.

**Figure 12:**
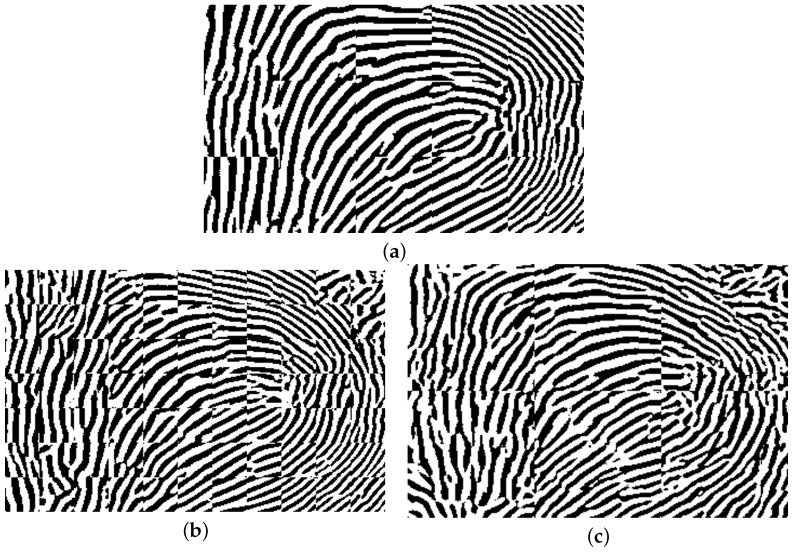
A sample enhanced fingerprint image by merging the enhanced patches shown in (**a**). The trained U-Net model was used to enhance the patches of size 64 pixels. A sample enhanced fingerprint image by merging the enhanced patches is shown in (**b**) with patch size 32, and in (**c**) with a patch size of 112 pixels.

The trained model can be used for fingerprint enhancement. First, the generated gray-scale fingerprints from the flattened point cloud were segmented into patches of 64 by 64 pixels. Next, the patches were enhanced by using the trained model. Lastly, the enhanced patches were merged. Figure 12a shows a sample enhanced fingerprint obtained by merging the enhanced fingerprint patches. Figure 12b,c show another two samples of enhanced fingerprint images by merging the enhanced patches with patch sizes of 32 and 112 pixels, respectively. It can be observed that the patch-based method successfully resolves the issue of blank areas. However, there is still the problem of misaligned ridge and valley patterns, which may result from enhancing the patches separately. This problem becomes more significant when using smaller patch sizes, such as 32 pixels. As the patch size decreases, more cuts are required to generate the patches, which increases the chance of misaligned ridge and valley patterns.

#### 2.2.3. U-Net-Based Full Image Fingerprint Enhancement by Using Quality Map

**Figure 13:**
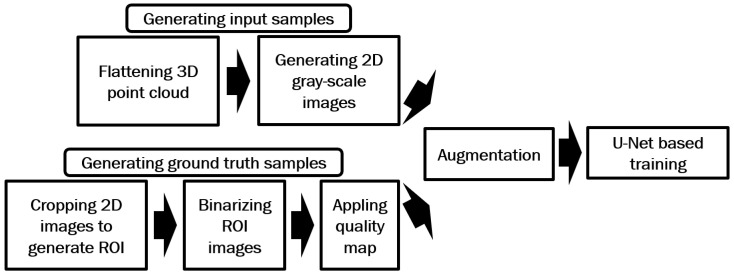
Processes used in the proposed method for generating the training data for U-Net-based training.

The objective of this method is to prevent blank spots in the enhanced image while ensuring that ridge and valley patterns remain properly aligned. To achieve this, we use high-quality ground truth images for training and utilize the entire image to avoid issues with discontinuous ridge and valley patterns. The same U-Net enhancement model as shown in Figure 6 is also used here, with an image input and output size of 352 by 248 pixels. Figure 13 illustrates the processes involved in the proposed method for generating training data.

**Figure 14:**
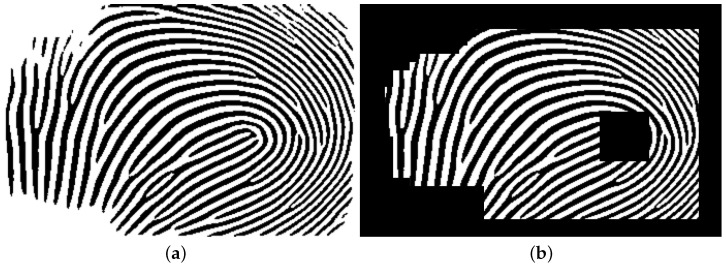
A sample binarized image from Figure 3a is shown in (**a**), and its quality-mapped image, which only includes the highest-quality sections of the binarized image (quality 4), is shown in (**b**).

Similar to the method described in Section 2.2.1, the full-size generated gray-scale images were used as input data for the U-Net model, while the binarized ROI images served as ground truth data. MINDTCT [20] was also applied to the binarized images in this approach. In contrast to the patch-based method, which used sections with quality levels of 2, 3, and 4 for training, this approach utilizes only the highest-quality sections (quality 4). The intensities of these sections were shifted to the range [1, 255], while the low-quality sections were assigned an intensity of zero. Figure 14a shows a sample binarized image from Figure 3a, and its quality-mapped image, which only includes the highest-quality sections of the binarized image (quality 4), is shown in Figure 14b. Finally, the input and ground truth images were augmented by a horizontal flip, vertical flip, and both a horizontal and vertical flip, resulting in a total of 5040 input and ground truth images.

**Figure 15:**
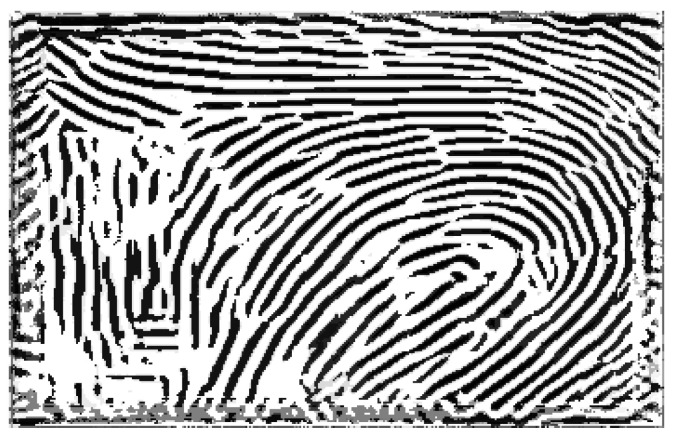
The enhanced image of the generated gray-scale image shown in Figure 1d, using the proposed method in Section 2.2.3.

**Figure 16:**
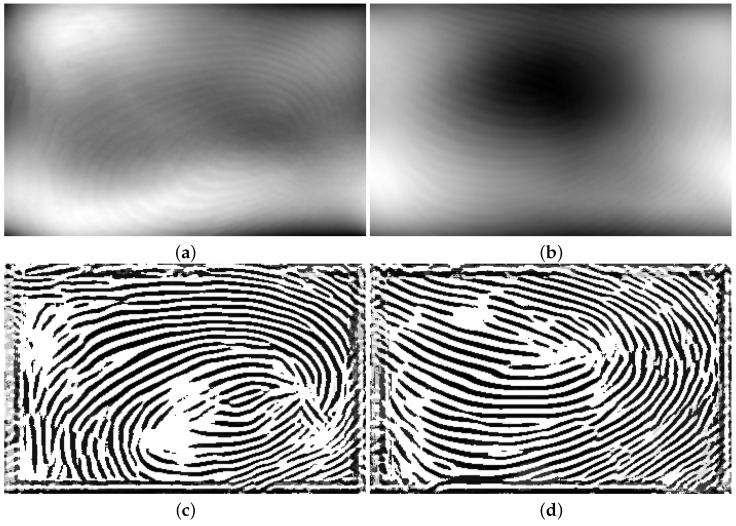
The 2D images and their corresponding ROI images of the first 3D impression of the first person in the first session of the dataset are shown in (**a**–**d**).

The model includes 256 classes to cover all potential intensity levels in a gray-scale image. Class zero is excluded since it is used by the quality map to indicate the low-quality regions of the generated ground truth. The training parameters are shown in Table 3. Figure 15 shows the gray-scale image generated from Figure 1d, enhanced using the proposed method. Figure 16a,b show two additional flattened 3D point cloud samples. The enhanced images of these samples are shown in Figure 16c and Figure 16d, respectively. A comparison between the enhanced images produced by the revised proposed method and those generated in the previous sections, shown in Figure 7b and Figure 12a, demonstrates that the proposed method significantly reduces the size of blank areas in the enhanced images while also preventing misaligned ridge and valley patterns.

## 3. Experimental Result

This section describes the tool setup and the evaluation of the proposed method for enhancing fingerprint images.

### 3.1. Dataset

To train the model and test it, the Hong Kong Polytechnic University 3D Fingerprint Images Database Version 2 [18,21] was used. The fingerprint impressions of the first 210 subjects in session 1 of this dataset were used for training the model (Dataset A), and the fingerprint impressions of the subjects 211 to 300 (Dataset B) were used for testing in the first experiment. Session 2 of this database has 200 subjects (Dataset C), which were used for testing in the second experiment. In both sessions, each subject had six 3D fingerprint impressions, which were represented in 3D point cloud format with the dimensions of 900 by 1400 points. For each point cloud impression, two corresponding contactless 2D images were also available, with dimensions of 1536 by 2048, which were captured with different illuminations.

### 3.2. Tools

The experiments were performed on a Dell workstation (Dell, Round Rock, TX, USA) with a 12th generation Intel Core i9 CPU, 32Gb of RAM (Intel, Santa Clara, CA, USA), and an NVIDIA GeForce RTX 3060 GPU (NVIDIA, Santa Clara, CA, USA). Microsoft Windows 11 [22], Matlab R2023b [23], PyCharm 2024 [24], and Visual Studio 2022 [25] were used for training the model and running the experiments. MINDTCT [20] was used to generate quality maps, and VeriFinger SDK 13.1 [19] was used to calculate fingerprint-matching scores.

### 3.3. Setup

This section explains the preparation of input images and ground truth for constructing the training dataset required to train the model, as well as the preparation of the testing dataset.

The input data were generated by converting the 3D point clouds of Dataset A to gray-scale images as described in Section 2.1. The data were augmented by adding a horizontal flip, vertical flip, and (horizontal and vertical) flip of each image to the training data to make a total of 5040 images.

Similar to the input data, the testing data were generated by converting the 3D point clouds of Dataset B and Dataset C to gray-scale images, with a total of 540 and 1200 images, respectively.

The ground truth images were generated using the method proposed in Section 2.2.3 and augmented in the same manner as the input images, resulting in a total of 5040 images. These input and ground truth images were used to train the U-Net model described in Section 2.2.3 and subsequently applied to enhance fingerprint images in our experiments.

Similar to the input data, the generated quality map images were augmented to make a total of 5040 images.

### 3.4. Experiment A

**Figure 17:**
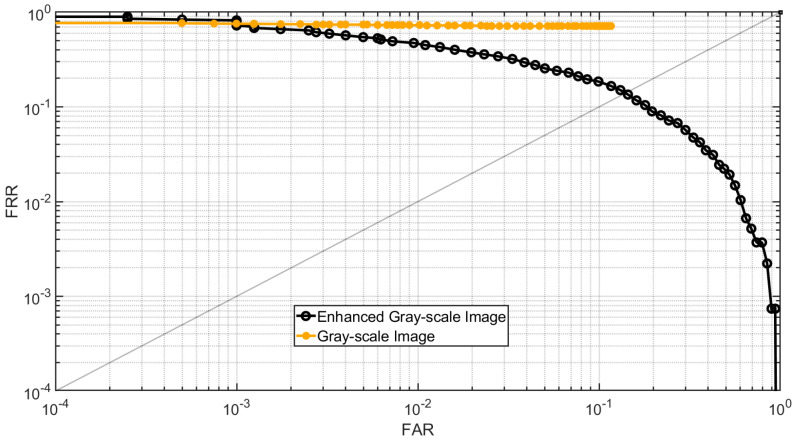
DET curve of gray-scale images and enhanced gray-scale images for Dataset B.

In the first experiment, the point clouds in Dataset B are first converted into gray-scale images using the method described in Section 2.1. Next, these images are enhanced using the proposed method detailed in Section 2.2.3. Following the FVC 2006 protocol, the genuine and imposter matching scores for the first impression of each subject are then calculated for both the gray-scale images and their enhanced versions. A total of 1350 genuine and 4005 imposter matching scores are obtained. Figure 17 presents the DET curve for the gray-scale images and their enhanced counterparts. The results indicate that the fingerprint recognition performance of the enhanced images has improved.

**Figure 18:**
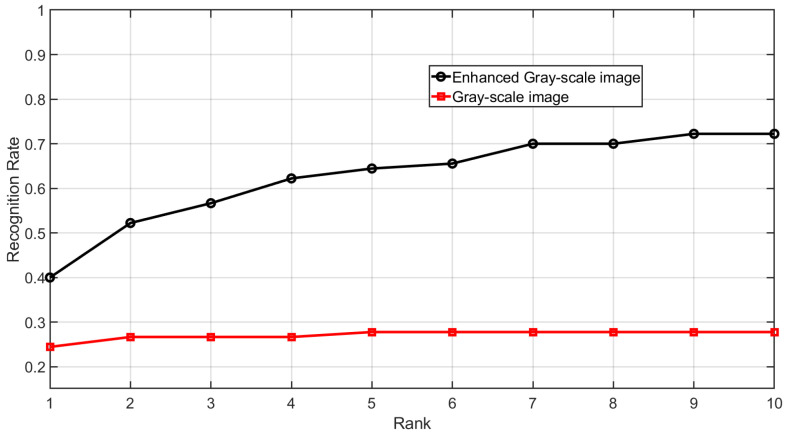
CMC curve of gray-scale images and enhanced gray-scale images for Dataset B.

The Cumulative Match Characteristic (CMC) curve is shown in Figure 18. It can be observed that the identification accuracy of the enhanced gray-scale images has improved. The CMC curve is generated by identifying each subject’s first impression within a gallery that includes the subject’s second impression, along with all impressions from 89 other subjects, resulting in a gallery size of 535 impressions.

Table 4 presents the calculated Equal Error Rate (EER), rank-1 accuracy, precision, recall, and F1-score for these experiments. The results show that fingerprint recognition performance and identification accuracy are improved for the images enhanced by the proposed method.

### 3.5. Experiment B

**Figure 19:**
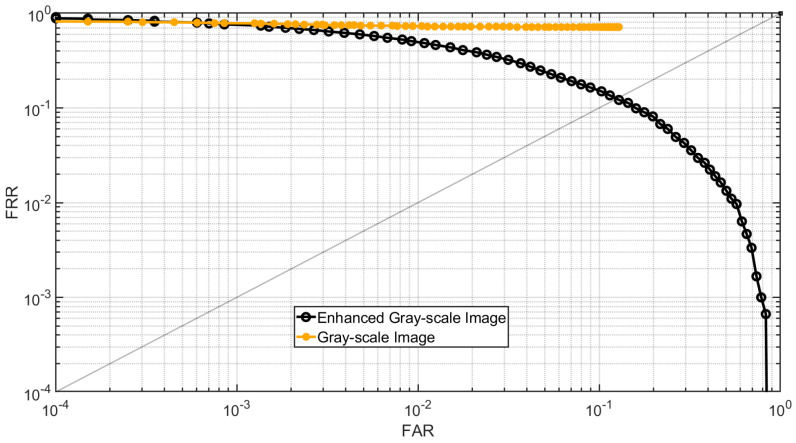
DET curve of gray-scale images and enhanced gray-scale images for Dataset C.

In the second experiment, Dataset C is used for testing. Similar to the previous experiment, gray-scale images are generated from the 3D point clouds using the method described in Section 2.1 and then enhanced using the proposed method detailed in Section 2.2.3. The genuine and imposter matching score for the gray-scale images and their enhanced images are calculated. For each case, there are 3000 genuine and 19,900 imposter matching scores. Figure 19 shows the DET curve of the gray-scale images and their enhanced counterparts using the proposed method. The results demonstrate that the enhanced fingerprint images improve fingerprint recognition performance.

**Figure 20:**
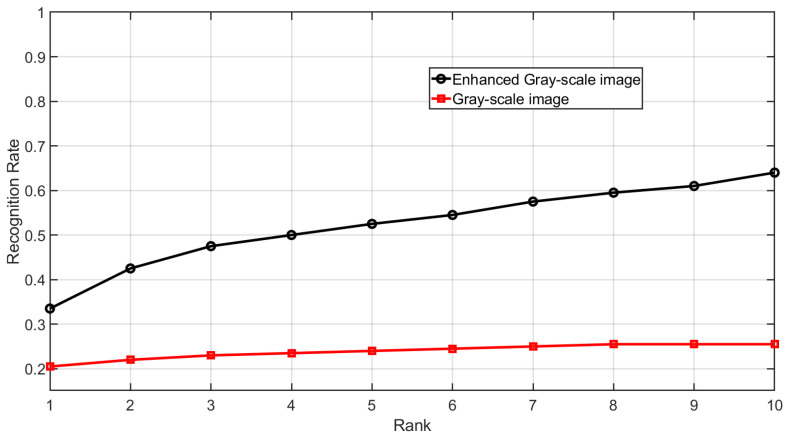
CMC curve of gray-scale images and enhanced gray-scale images for Dataset C.

Figure 20 shows the CMC curve of gray-scale images and their enhanced counterpart. It can be observed that the fingerprint identification accuracy of the enhanced fingerprint images is improved compared to their gray-scale counterparts. The CMC curve is obtained by identifying each subject’s initial impression within a gallery that includes the subject’s second impression along with all impressions from 199 other subjects, resulting in a gallery size of 1195 impressions.

Table 5 shows the calculated EER, rank-1 accuracy, precision, recall, and F1-score of these experiments. The results of this experiment also show that fingerprint recognition performance and identification accuracy are improved for the images enhanced by the proposed method.

### 3.6. Evaluation

In both experiments, the enhanced fingerprints achieved a lower EER and a higher rank-1 accuracy. These results indicate that the proposed fingerprint enhancement method is effective in improving recognition and identification performance, particularly when the fingerprints are of very low quality.

## 4. Conclusions

In this work, a modified 3D point cloud unwrapping method was implemented to flatten a 3D point cloud, which was then converted into gray-scale images. Since image intensities were derived from relative point heights in the point cloud, a specialized enhancement method was necessary, which this study addresses.

To improve image quality, three U-Net training approaches based on image pixel classification were proposed.

1. Full image enhancement;
2. Patch-based enhancement;
3. Filtered full-size image enhancement.

Each approach aimed to overcome the limitations of the previous method, with the final approach being examined and evaluated. Experimental results demonstrate that the proposed method effectively enhances low-contrast fingerprint images, leading to improved fingerprint recognition and identification performance.

While the third proposed approach serves as a better alternative to address the weaknesses of the first two methods, each approach requires further in-depth study and evaluation. Our future work will focus on resolving misaligned ridge and valley patterns in the enhanced images of the patch-based method, as this refinement has the potential to significantly improve performance and will be our primary research focus moving forward.

## Figures and Tables

**Table 1:** Parameters used in U-Net training.

**Loss Function**	Sparse Categorical Crossentropy
**Optimizer**	Adam (learning rate:0.001)
**Batch Size**	16
**Epochs**	320
**Number of Classes**	256

**Table 2:** Parameters used in patch-based U-Net training.

**Loss Function**	Sparse Categorical Crossentropy
**Optimizer**	Adam (learning rate:0.001)
**Batch Size**	16
**Epochs**	100
**Number of Classes**	2

**Table 3:** Parameters used in U-Net training.

**Loss Function**	Sparse Categorical Crossentropy
**Optimizer**	Adam (learning rate:0.001)
**Batch Size**	16
**Epochs**	1500
**Number of Classes**	255

**Table 4:** Comparison of equal error rate, rank-1 accuracy, precision, recall, and F1-score of the generated gray-scale images by flattening the 3D point cloud and their enhanced fingerprint images for Dataset B using the proposed method.

Experiments	EER	Rank-1 Accuracy	Precision	Recall	F1-Score
Generated gray-scale images from the flattened point cloud	41.32%	24.44%	45.68%	28.96%	35.45%
Enhanced gray-scale images	13.96%	40.00%	66.90%	86.52%	75.45%

**Table 5:** Comparison of equal error rate, rank-1 accuracy, precision, recall, and F1-score of the generated gray-scale images by flattening the 3D point cloud and their enhanced fingerprint images for Dataset C using the proposed method.

Experiments	EER	Rank-1 Accuracy	Precision	Recall	F1-Score
Generated gray-scale images from the flattened point cloud	41.97%	20.5%	25.33%	28.90%	27.00%
Enhanced gray-scale images	12.49%	33.5%	50.74%	87.87%	64.33%

## Data Availability

Data are contained within the article.
